# Trends in Life Expectancy in Residential Long-Term Care by Sociodemographic Position in 1999–2018: A Multistate Life Table Study of Finnish Older Adults

**DOI:** 10.1093/geronb/gbae067

**Published:** 2024-04-17

**Authors:** Kaarina Korhonen, Heta Moustgaard, Michael Murphy, Pekka Martikainen

**Affiliations:** Helsinki Institute for Demography and Population Health, Faculty of Social Sciences, University of Helsinki, Helsinki, Finland; Max Planck—University of Helsinki Center for Social Inequalities in Population Health, Faculty of Social Sciences, University of Helsinki, Helsinki, Finland; Helsinki Institute for Demography and Population Health, Faculty of Social Sciences, University of Helsinki, Helsinki, Finland; Helsinki Institute for Social Sciences and Humanities (HSSH), University of Helsinki, Helsinki, Finland; Department of Social Policy, London School of Economics and Political Science, London, UK; Helsinki Institute for Demography and Population Health, Faculty of Social Sciences, University of Helsinki, Helsinki, Finland; Max Planck—University of Helsinki Center for Social Inequalities in Population Health, Faculty of Social Sciences, University of Helsinki, Helsinki, Finland; (Social Sciences Section)

**Keywords:** Education, Marital status, Mortality, Nursing home

## Abstract

**Objectives:**

Residential long-term care (LTC) use has declined in many countries over the past years. This study quantifies how changing rates of entry, exit, and mortality have contributed to trends in life expectancy in LTC (i.e., average time spent in LTC after age 65) across sociodemographic groups.

**Methods:**

We analyzed population-register data of all Finns aged ≥65 during 1999–2018 (*n* = 2,016,987) with dates of LTC and death and sociodemographic characteristics. We estimated transition rates between home, LTC, and death using Poisson generalized additive models, and calculated multistate life tables across 1999–2003, 2004–2008, 2009–2013, and 2014–2018.

**Results:**

Between 1999–2003 and 2004–2008, life expectancy in LTC increased from 0.75 (95% CI: 0.74–0.76) to 0.89 (95% CI: 0.88–0.90) years among men and from 1.61 (95% CI: 1.59–1.62) to 1.83 (95% CI: 1.81–1.85) years among women, mainly due to declining exit rates from LTC. Thereafter, life expectancy in LTC decreased, reaching 0.80 (95% CI: 0.79–0.81) and 1.51 (95% CI: 1.50–1.53) years among men and women, respectively, in 2014–2018. Especially among women and nonmarried men, the decline was largely due to increasing death rates in LTC. Admission rates declined throughout the study period, which offset the increase in life expectancy in LTC attributable to declining mortality in the community. Marital status differences in life expectancy in LTC narrowed over time.

**Discussion:**

Recent declines in LTC use were driven by postponed LTC admission closer to death. The results suggest that across sociodemographic strata older adults enter LTC in even worse health and spend a shorter time in care than before.

The use of residential long-term care (LTC) increases with advancing age, particularly in the years preceding death (Larsson et al., 2008; Martikainen et al., 2012; Murphy & Martikainen, 2013). As life expectancy rises, the demand for LTC is expected to grow, imposing significant strain on welfare state care sectors and the families of older adults.

In anticipation of the heightened demand for LTC, several countries have undergone substantial reforms in their LTC services since the early 1990s (Pavolini & Ranci, 2008). In Finland, where the proportion of population aged 65 and over ranks fourth highest among Organization for Economic Cooperation and Development (OECD) countries, adjustments to LTC provision have entailed a gradual retrenchment of LTC provision. Traditional institutions like nursing homes have been replaced by service housing with round-the-clock assistance (Anttonen & Karsio, 2016). Simultaneously, deliberate efforts have been made to shift the emphasis from LTC towards community care. With a more stringent focus on targeting LTC to those most in need, the eligibility criteria for LTC have become stricter over time (Rostgaard et al., 2022). Consequently, the proportion of older adults over age 75 residing in LTC has decreased by about a third from 15% in 1990 to 10% in 2015 (Kröger, 2019). This declining coverage aligns with an international trend observed in various OECD countries with relatively generous public LTC provisions, including the Netherlands, Switzerland, Ireland, New Zealand, and other Nordic countries (OECD, 2023).

Increasing life expectancy and reforms in LTC policies are crucial influences on changes in LTC entry/exit rates and mortality, which determine the shifting life expectancy in LTC (i.e., the years that individuals can anticipate residing in LTC). A decrease in admission rates can influence life expectancy in LTC by reducing the proportion of older adults entering LTC and potentially shortening the duration of LTC residence for those who do enter. Previous research from the Netherlands suggests that the decline in admission rates to LTC is predominantly associated with institutional changes or shifts in preferences rather than improvements in health and disability (Alders et al., 2017; De Meijer et al., 2015). However, the overall decline in mortality simultaneously increases the proportion of older adults surviving to ages where LTC entry becomes more likely. Consequently, the contributions of improving mortality to life expectancy in LTC remain unclear.

The duration of LTC residence is further influenced by exits, either through returning to the community or dying in LTC. The death rate, in particular, is relevant as individuals entering LTC commonly spend their final years there (Martikainen et al., 2009). Some studies suggest an increasing risk of death in LTC over time (Grundy, 2011; Pujol et al., 2021; Sund Levander et al., 2016), indicating a possible deterioration in the health status of LTC residents due to increasingly stringent admission criteria. To our knowledge, no prior study has quantified the combined effects of changing LTC entry and exit rates and declining mortality (in the community) on recent LTC trends at the population level or estimated the contributions of each factor.

In addition to age, various sociodemographic factors including sex, marital status, and education are important determinants of both mortality and LTC use. Notably, women outlive men (Zarulli et al., 2021) and are more likely to enter LTC (Martikainen et al., 2009). Conversely, nonmarried individuals and those with lower educational attainment tend to live shorter lives (Kaplan & Kronick, 2006; Mackenbach et al., 2019) but are more likely to enter LTC compared to those who are married or have higher education (Martikainen et al., 2014; Nihtilä & Martikainen, 2007). Sociodemographic groups have also experienced differential changes in life expectancy, with larger gains observed among men (Cui et al., 2019), those who are married (Peltonen et al., 2017), and individuals with higher educational attainment (van Raalte et al., 2018). Although one Dutch study reported similar declines in the likelihood of LTC use for both men and women (Aaltonen et al., 2022), there is currently a lack of prior evidence showing how life expectancy in LTC has evolved across different sociodemographic groups. A notable challenge in the existing literature is the lack of population-level data, which can hinder the generalizability of findings, potentially overlooking important trends and variations across diverse populations.

Leveraging longitudinal population-level register data, our study explores the changing use of LTC overall and in population subgroups, by analyzing trends in life expectancy in LTC by sex, marital status, and educational level from 1999 to 2018. We also investigate changes in the proportion of 65-year-olds ever entering LTC, the median age at first entry, and the duration spent in LTC among those who enter. Finally, our analysis quantifies the contributions of shifting rates of LTC entry, exit, and mortality (in the community) to observed changes in life expectancy in LTC. This detailed exploration aims to improve our understanding of the various factors influencing changing patterns of LTC use, guiding future research endeavors, providing valuable insights for policy, and assisting individuals and families in making informed decisions about LTC.

## Method

### Data and Study Population

We used population register data of all Finnish residents between 1999 and 2018. We restricted the analysis to the population aged ≥65 years. People who turned 65 in 1999–2018 contributed to the person-years and transitions between home, LTC, and death beginning on the first day of the month of their 65^th^ birthday. The population register maintains records of the population resident in Finland on December 31 each year, and thus individuals were followed up each year conditional on Finnish residency at the end of the previous year. After exclusions, the total cohort included 2,016,987 individuals. Statistics Finland linked the population registers with the Death Register for dates of death and the Registers of Health and Social Care collected by the Finnish Institute for Health and Welfare for dates in LTC.

The study was approved by the Statistics Finland Board of Ethics and the Social and Health Data Permit Authority Findata (permit numbers TK/3343/07.03.00/2023 and THL/499/14.06.00/2024). Statistics Finland pseudonymized the data prior to providing it to researchers.

#### Residential long-term care

We extracted LTC residence periods that began or ended between January 1, 1999 and December 31, 2018 from the Care Register of Health and Social Care. This register, mandated by the Act on the National Institute for Health and Welfare, meticulously compiles data from both public and private social and healthcare service providers. LTC was defined as stays in nursing homes, health centers, hospitals, service housing with 24-h assistance, and rehabilitation that lasted for at least 90 days or were confirmed by an administrative LTC decision. If a person received care at multiple sites or there was a maximum of one night between residence periods, we considered these as one continuous spell. We included up to 20 LTC residence spells since having more than this was very rare (*n* = 147). Most individuals with LTC (77%) only had one residence period from 1999 to 2018. The exit date was unavailable for 4.2% of LTC spells. In these cases, we set the exit date as January 1, which was the following day of the end-of-year patient census when the person was known to be present.

#### Sociodemographic characteristics

Information on sex, marital status, and educational level were obtained from the population register of Statistics Finland. Marital status and educational level were updated on December 31 each year. Marital status distinguished married, widowed, divorced, and never married individuals. Nonmarital cohabitation was not specifically examined since, by definition, it is not possible for individuals in LTC. However, given the infrequency of nonmarital cohabitation among older adults (Moustgaard & Martikainen, 2009), the recorded marital status serves as a reasonable proxy for living arrangements. Education was based on the highest attained qualification, categorized as high (International Standard Classification of Education ISCED-2011 5–8), intermediate (ISCED 3–4), and low education (ISCED 0–2).

### Statistical Analysis

We applied the multistate life table framework, a well-suited method for analyzing complex transitions among multiple states. In our application, we simulated the dynamic progression of the Finnish population aged 65 and over through various states, including living at home, residing in LTC (transient states), and death (an absorbing state). The transitions between these states are determined by age-specific probabilities obtained from the empirical data and estimated separately for population strata of interest.

For descriptive purposes, we first estimated total life expectancy and life expectancy in LTC at age 65 by sex and year in 1999–2018. To analyze changes in more detail, we used broader time periods (1999–2003, 2004–2008, 2009–2013, and 2014–2018) to reduce random variation due to small numbers of age-specific transitions in each calendar year. The sociodemographic distribution of the study population and the number of transitions to LTC in each period are shown in Supplementary Table 1.

We initially estimated transition rates between the three states based on observed transitions per person-year at risk in 0.1-year age groups. Transition rates were estimated using Poisson generalized additive models (GAM) with smooth functions for age and stratified by sex and period. Conditional transition probabilities were then calculated as in traditional life tables (*q*_*x*_^*t*^ = 1-*exp*(*-m*_*x*_^*t*^)), where *m*_*x*_^*t*^ is the transition rate at age *x*, and *t* is the time scale.

We present results for synthetic cohorts starting at exact age of 65, initially distributed between home and LTC according to the prevalence of LTC at exact age of 65 years modeled using logistic regression with continuous age (in years), age-squared, and age-cubed as covariates. The synthetic cohort was then updated in the life table using the estimated age-specific transition probabilities up to age 105 years.

We calculated the median age at first entry, the proportion of 65-year-olds ever entering, and years in LTC among those who entered. The median age at first entry was defined as the age by which 50% of those who will ever enter LTC have done so. The proportion ever entering LTC was calculated as the proportion of the initial life table cohort that started in or entered LTC. Years in LTC among those who entered was then defined as the total life table years spent in LTC divided by the number of individuals starting in or entering LTC. In calculating these three parameters, we incorporated transition rates that only considered the first entry to LTC for each person in the respective period.

We estimated the contribution of changes in each type of transition (home ➔ LTC, home ➔ death, LTC ➔ home, and LTC ➔ death) to the total change in life expectancy in LTC by holding the transition probabilities for each type constant at the values observed in the previous 5-year period while allowing all other transition probabilities to change. Since these four types of contributions determine the total change in life expectancy in LTC over time, this is in practice a decomposition analysis. The home ➔ LTC transition reflects changes in policy or preferences, or in health. Changes in the home ➔ death transition reflect the overall mortality decline in the population. The LTC ➔ home transition reflects changes in health-related factors (e.g., changing disability within LTC or rehabilitation) or institutional factors (e.g., changes in community care services). Finally, changes in the LTC ➔ death transition are associated with a changing level of ill-health or disability among LTC residents. Age-specific annual transition probabilities between home, LTC, and death for each period are presented in Supplementary Figures 1 and 2.

Finally, we estimated multistate life tables by marital status and education. Confidence intervals (CI) were obtained by bootstrapping with 500 resamples drawn. Supplementary analyses estimated life expectancy in LTC separately for the Helsinki capital region, other large cities, and the rest of Finland, to see if the trends were similar for more and less densely populated areas. All analyses were conducted using R version 4.0.5.

## Results

**Figure 1.**
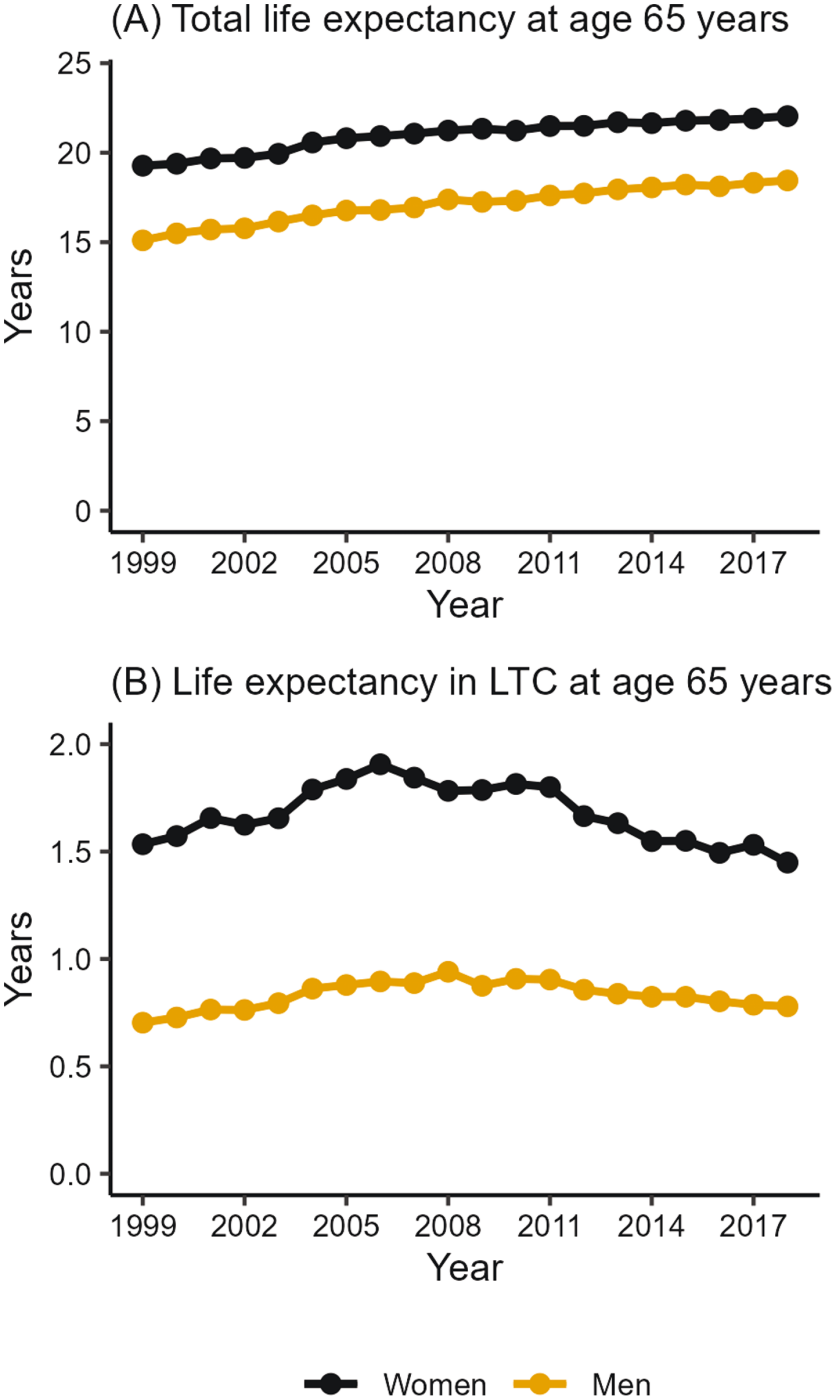
Total life expectancy (**A**) and life expectancy in residential long-term care (LTC) (**B**) at age 65 years in 1999–2018, by sex.

Total life expectancy at age 65 years increased from 15.10 (95% CI: 14.99–15.21) to 18.45 (95% CI: 18.35–18.55) years for men and from 19.27 (95% CI: 18.18–19.37) to 22.04 (95% CI: 21.95–22.13) years for women between 1999 and 2018 (Figure 1). These estimates match well with those of the Official Statistics of Finland (Supplementary Table 2). From 1999, life expectancy in LTC increased until 2006 for women (1.91; 1.87–1.94 years) and 2008 for men (0.94; 0.91–0.98 years). Thereafter, life expectancy in LTC declined rather steadily, reaching 1.45 (1.42–1.48) years for women and 0.78 (0.76–0.80) years for men in 2018.

Table 1 shows the multistate life table results for periods 1999–2003, 2004–2008, 2009–2013, and 2014–2018. Life expectancy in LTC increased by 0.14 (0.12–0.16) years for men and 0.22 (0.20–0.24) years for women between 1999–2003 and 2004–2008. The subsequent decline was especially large between 2009–2013 and 2014–2018 (−0.07 [−0.09 to −0.06] years for men and −0.23 [−0.24 to −0.20] years for women). Although the median age at first entry increased consistently across all the periods, the proportion ever entering and years in LTC for those who entered increased from 1999–2003 to 2004–2008 and declined thereafter.

**Table 1.** Multistate Life Table Results for Residential Long-term Care (LTC) at Age 65 Years, by Sex and Period, Finnish Men and Women in 1999–2018

Sex	Period	Life expectancy in LTC^a^		% Ever entering	Median age at first entry (years)	Years in LTC if entered
		Years	Change from previous period, years			
Men						
	1999–2003	0.75 (0.74–0.76)		33.5 (33.2–33.8)	80.9 (80.8–81.0)	2.24 (2.21–2.27)
	2004–2008	0.89 (0.88–0.90)	0.14 (0.12 to 0.16)	35.1 (34.8–35.4)	82.2 (82.1–82.3)	2.53 (2.49–2.58)
	2009–2013	0.87 (0.86–0.89)	−0.02 (−0.03 to 0.00)	35.1 (34.8–35.4)	83.0 (82.9–83.1)	2.49 (2.45–2.53)
	2014–2018	0.80 (0.79–0.81)	−0.07 (−0.09 to −0.06)	33.8 (33.5–34.1)	84.0 (83.9–84.1)	2.37 (2.33–2.40)
Women						
	1999–2003	1.61 (1.59–1.62)		51.3 (51.0–51.6)	83.3 (83.2–83.3)	3.14 (3.10–3.17)
	2004–2008	1.83 (1.81–1.85)	0.22 (0.20 to 0.24)	52.3 (52.0–52.6)	84.5 (84.4–84.6)	3.50 (3.46–3.53)
	2009–2013	1.74 (1.72–1.75)	−0.09 (−0.12 to −0.07)	51.6 (51.3–51.9)	85.3 (85.3–85.4)	3.37 (3.33–3.40)
	2014–2018	1.51 (1.50–1.53)	−0.23 (-0.24 to −0.20)	48.9 (48.6–49.2)	86.2 (86.1–86.3)	3.09 (3.06–3.13)

*Note*:

^a^Years of total life expectancy at age 65 years spent in LTC.

**Figure 2.**
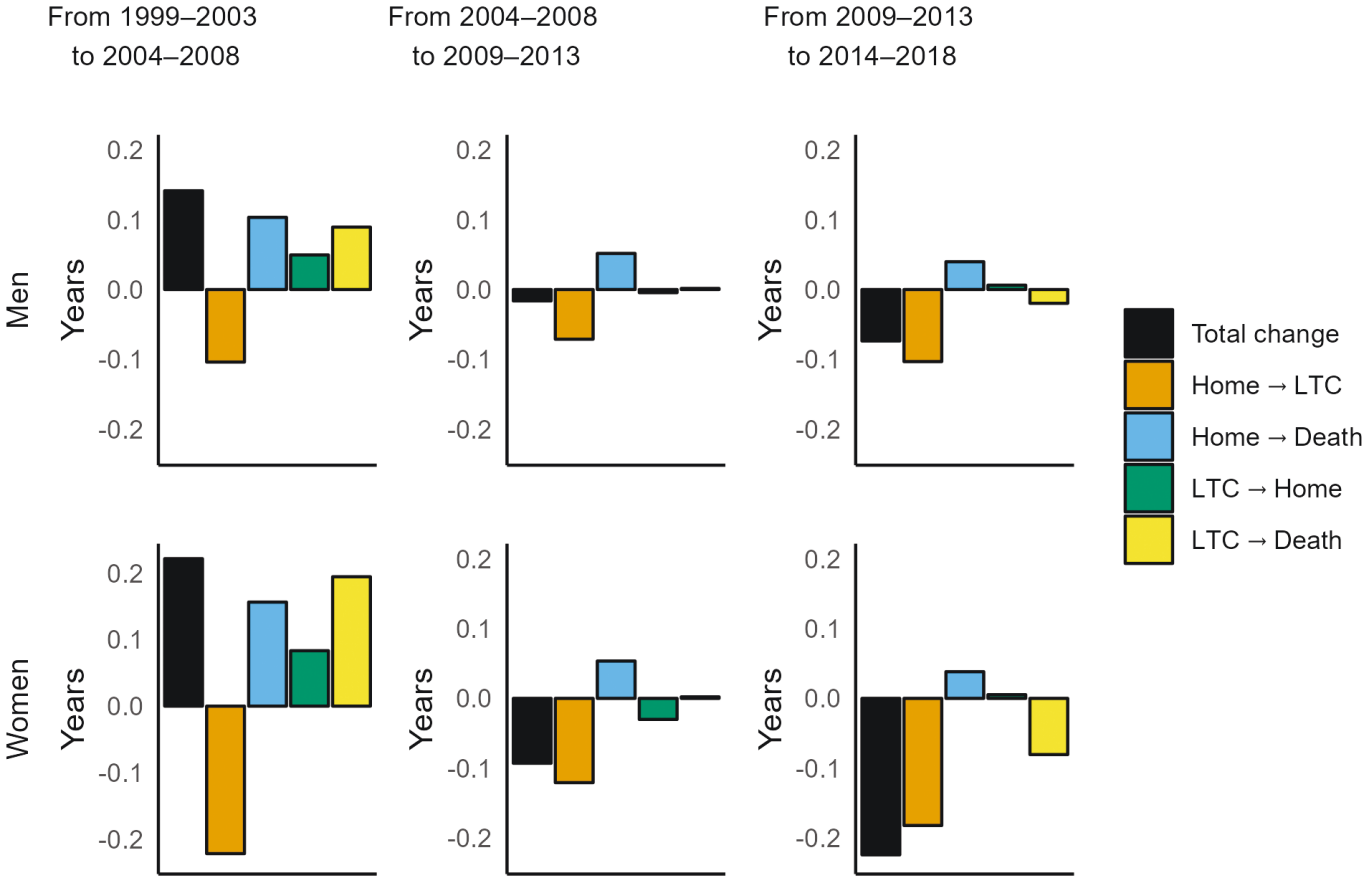
Contributions of changes in transition rates between home, residential long-term care (LTC), and death to the change in life expectancy in LTC at age 65 years between 1999–2003, 2004–2008, 2009–2013, and 2014–2018, by sex.

Admission rates declined in all periods and contributed to a decline in life expectancy in LTC (Figure 2; Supplementary Figures 1 and 2). By contrast, declining mortality in the community contributed to an increase in life expectancy in LTC, but this was fully offset by declining admission rates in all periods. The increase in life expectancy in LTC from 1999–2003 to 2004–2008 was thus mainly attributable to substantial declines in the LTC exit rate through returning to the community or dying. The modest decline in life expectancy in LTC from 2004–2008 to 2009–2013 was mainly related to the continued decline in admission rates. Between 2009–2013 and 2014–2018, the declining admission rate was combined with an increase in the LTC death rate, which especially among women contributed to the large declines in life expectancy in LTC.

**Figure 3.**
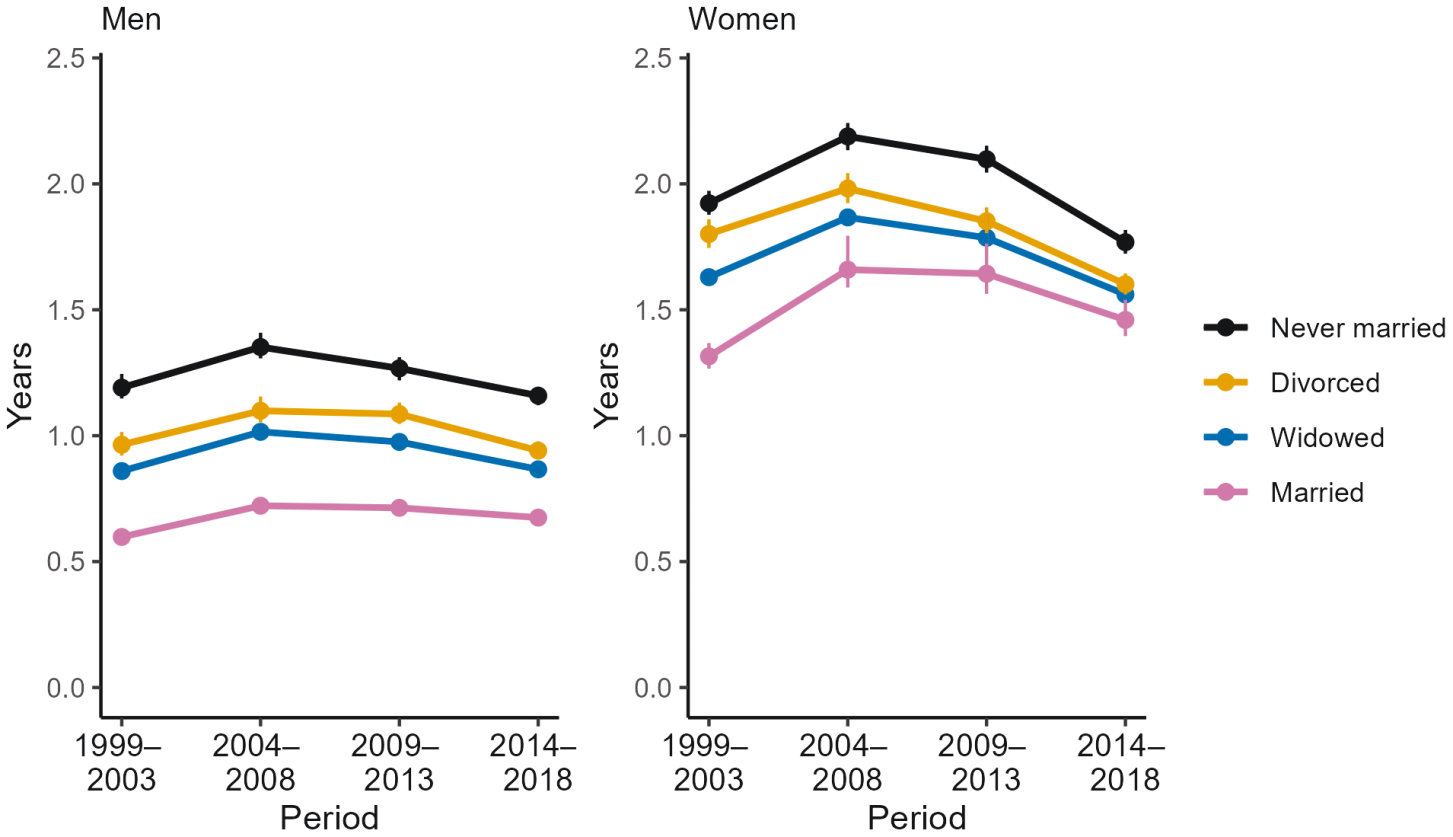
Life expectancy in residential long-term care (LTC) at age 65 years, by marital status and period, and by sex.

Married individuals had the shortest life expectancy in LTC throughout the study period (Figure 3; Supplementary Tables 3 and 4). The differentials narrowed across time, however, as the recent declines in life expectancy in LTC were more pronounced for the nonmarried individuals. Supplementary Figure 3 shows that the decline in life expectancy in LTC among nonmarried men was attributable to larger declines in admission rates and increasing LTC death rates compared to married men. Among women, by contrast, declining admission rates and increasing LTC death rates contributed to a reduced life expectancy in LTC in all marital status groups, but declining mortality in the community attenuated these effects for the married (Supplementary Figure 4).

**Figure 4.**
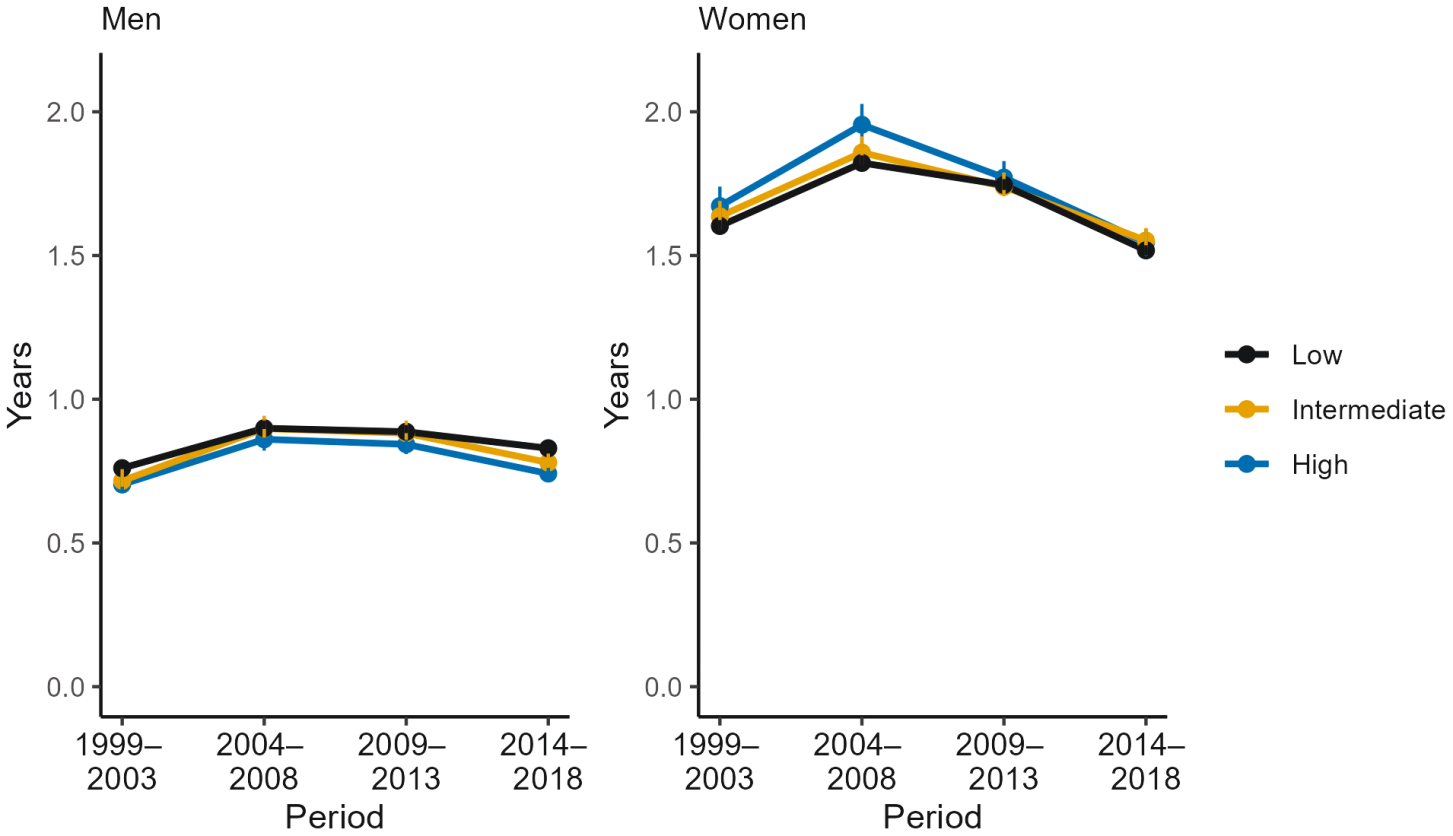
Life expectancy in residential long-term care (LTC) at age 65 years, by educational level and period, and by sex.

Educational differences in life expectancy in LTC were small throughout the study period (Figure 4, Supplementary Tables 3 and 4). For both men and women, the proportion ever entering LTC was higher for the more highly educated across the study periods (Supplementary Table 4).

Supplementary analysis showed that the somewhat higher level of life expectancy in LTC observed in the Helsinki capital region compared to other large cities and the rest of Finland in 1999–2003 and 2004–2008 disappeared in the more recent periods (Supplementary Figure 7), as the postponement of LTC admissions closer to death began earlier in the Helsinki metropolitan area (Supplementary Figures 8 and 9).

## Discussion

Life expectancy in LTC at age 65, representing the time that a 65-year-old can expect to live in LTC in their remaining lifetime, saw an increase in Finland between 1999–2003 and 2004–2008. This rise in life expectancy in LTC occurred despite declining admission rates and was mainly attributable to decreasing LTC exit rates through returning to the community or dying. Subsequently, there was a notable decline in life expectancy in LTC, especially from 2009–2013 to 2014–2018. This decline was related to sustained declines in admission rates and, particularly among women and nonmarried men, to increased LTC death rates.

Our findings demonstrate that even in contexts where eligibility criteria for LTC are tightened, a decline in admission rates does not necessarily translate to a reduction in overall (per capita) use of LTC, as indicated by life expectancy in LTC. On the contrary, life expectancy in LTC witnessed an increase in Finland in the early 2000s, mainly attributed to a decline in the LTC exit rate. A decrease in the exit rate at all ages implies that the likelihood of returning to the community or dying in LTC was lower than before, leading to an extended duration of care for those entering LTC. This shift is likely influenced by health-related factors. Prior studies have reported an increase in LTC use at the end of life in Finland during the same period (Aaltonen et al., 2019; Forma et al., 2017), primarily due to a growing proportion of individuals aged 80 and older with dementia (Aaltonen et al., 2019). Dementia, a prominent cause of disability in older populations, is associated with extended length of LTC stay at the end of life (Korhonen et al., 2018; Martikainen et al., 2012). Relatedly, consistent increases in the proportion of LTC residents living with dementia have been reported in Finland (Finne-Soveri et al., 2014) and elsewhere (Green et al., 2017; Matthews et al., 2013). The declining exit rate and associated increase in life expectancy in LTC in the early 2000s are likely linked to the increasing proportion of LTC residents experiencing dementia-related disability.

Substantial declines in life expectancy in LTC were only observed from 2009–2013 to 2014–2018, when the decline in admission rates was linked to an increase in the risk of death in LTC, suggesting a postponement of LTC entry closer to the end of life. An increase in the LTC death rate implies that the population residing in LTC is in poorer health than before, though in some contexts it might also suggest deterioration in the quality of care over time. Similar increasing trends in LTC death rates have also been reported in England and Wales (Grundy, 2011; Pujol et al., 2021) and Sweden (Sund Levander et al., 2016), although starting earlier than observed in Finland. Concurrently, there has been a surge in the level of disability among community care recipients. In Finland, the proportion of community care recipients with cognitive problems increased from about 60% in 2008 to 75% in 2018 (Finne-Soveri et al., 2014; The Finnish Institute for Health and Welfare, 2022a). Additionally, the proportion diagnosed with dementia doubled from less than 10% to more than 20% between 2001 and 2015 (Official Statistics of Finland (OSF), 2017; The Finnish Institute for Health and Welfare, 2023). Reflecting our results against these trends, the recent decline in life expectancy in LTC appears to be associated with an elevated access threshold, even for people with severe disabilities like dementia. Given the concurrent shortage of LTC places for those assessed as eligible (The Finnish Institute for Health and Welfare, 2022b), these trends cast doubt on the fulfillment of care needs.

The observed trends in Finland likely reflect comparable patterns in other Nordic countries, collectively characterized by the “public service model” (Anttonen & Sipilä, 1996). These nations have undergone similar retrenchment of LTC services, leading to parallel declines in the prevalence of LTC residence, as documented in prior studies (Rostgaard et al., 2022). In contrast, countries relying more extensively on informal care models, where shifts in public provision of LTC play a smaller role, may experience a stronger contribution of mortality decline to an increase in life expectancy in LTC. This hypothesis warrants examination using appropriate international data sets.

Although previous reports (Martikainen et al., 2014) have shown that nonmarried individuals had a longer life expectancy in LTC than their married counterparts, our study reveals a narrowing of these differentials over time. The recent decline in life expectancy in LTC, attributed to the postponement of LTC closer to death, was evident across all marital status groups among women but only observed for nonmarried men. Possible compositional changes in terms of, for example, co-habiting status and the availability of adult children, might have augmented the supply of informal care within nonmarried groups, potentially contributing to the delayed LTC admission. Similarly, the observed change among married women could be linked to an improved health status of husbands to serve as carers. Disentangling to what extent the postponement of LTC admissions among nonmarried men and women, as well as married women, relates to greater availability of informal care versus more stringent eligibility criteria for LTC placement requires future research.

Despite significant disparities in total life expectancy between educational groups, our study reveals modest educational differences in life expectancy in LTC across the study period. Factors more directly linked to material resources that facilitate independent living, such as wealth, income, or housing conditions, might be stronger predictors of LTC admission than education (Nihtilä & Martikainen, 2007). Notably, our findings highlight that the lifetime probability of entering LTC was higher for individuals with higher educational attainment compared to those with low education.

This study utilized unique Finnish total population register data and thus also covered institutionalized and socioeconomically marginalized groups and those experiencing health problems; groups that are difficult to reach in prospective cohort studies. Thus, the results were not affected by self-selection bias or selective attrition. Importantly, the linked data from care registers—with exact dates of entries to and exits from LTC—enabled consistent measurement of LTC use across two decades. Transitions between home, LTC, and death were pooled over 5-year periods, and therefore any yearly variation of, for example, influenza epidemics or heat waves were unlikely to affect our estimates. This is especially relevant for interpreting the results regarding the risk of death in LTC. Furthermore, the effects of COVID-19 do not affect our results because the follow-up was restricted to years before the pandemic.

Life table calculations present results for population subgroups assuming that people stay in the same group throughout their lives. This is a plausible assumption for attained education, but not for marital status as people marry and divorce throughout their lives, and especially the rate of becoming widowed increases with age. Therefore, the results—like all life expectancy estimates—express the hypothetical scenario where constant group-specific transition rates are assumed. The information on marital status was, however, updated each calendar year, thus allowing the correct allocation of person-years at risk and transitions from one state to another between marital statuses, and therefore any further bias from misclassification is minimal.

We do not have data on LTC needs. In the context of limited LTC places, for example, nonmarried older adults might be prioritized on waiting lists over married individuals who have a spouse living at home. In such a situation, the shorter life expectancy in LTC of the married would not only reflect lower care needs but higher thresholds for access to care. Relatedly, we cannot conclude to what extent the declines in LTC admission rates over time reflect improvements in health and disability or a heightened threshold for access to LTC. Previously, studies from the Netherlands have concluded that declines in admission rates in the early 2000s were attributable to institutional changes, including tightened eligibility criteria rather than to a decreased level of disability (Alders et al., 2017; De Meijer et al., 2015). To determine the extent to which institutional changes explain trends in life expectancy in LTC more recently and in Finland, further investigation is warranted.

Finally, we had no information on the use of community-based care for older adults. Especially in contexts where long-term care provided in the home is increasingly common, analysis of the length and intensity of community care before transitioning to LTC would complement the results of the current study.

In conclusion, the recent declines in life expectancy in LTC were driven by the postponement of LTC admission closer to death. Given the increasing burden of dementia-related disability associated with the postponement of mortality to older ages, it is unlikely that LTC admissions can be endlessly delayed closer to death while at the same time ensuring a good quality of life for older adults with serious functional and cognitive disabilities and their carers. Postponed LTC entry and prolonged time spent at home with serious disability also have implications for community care service provision and funding. Therefore, it is indispensable for welfare states with aging populations to ensure adequate resources for LTC provision.

## Supplementary Material

gbae067_suppl_Supplementary_Materials
