# Supplementary material for: Trends in Life Expectancy in Residential Long-Term Care by Sociodemographic Position in 1999–2018: A Multistate Life Table Study of Finnish Older Adults
Source: J Gerontol B Psychol Sci Soc Sci. 2024 Apr 17;79(7):gbae067. doi: 10.1093/geronb/gbae067 (PMC11157624; doi:10.1093/geronb/gbae067)
Supplement: gbae067_suppl_Supplementary_Materials [file gbae067_suppl_supplementary_materials.pdf]

Supplementary Material

Trends in Life Expectancy in Residential Long-Term Care by Sociodemographic Position in 1999–2018: A Multistate Life Table Study of Finnish Older Adults

Supplementary Table 1. Distribution of the study population and number of transitions to LTC by age, marital status and educational level, by sex and period

Subgroup	1999–2003 Person-years (%)	1999–2003 Transitions to LTC	2004–2008 Person-years (%)	2004–2008 Transitions to LTC	2009–2013 Person-years (%)	2009–2013 Transitions to LTC	2014–2018 Person-years (%)	2014–2018 Transitions to LTC
**Men**								
Age								
65–74	969,687 (63.8)	10,876	1,046,180 (60.7)	9745	1,225,546 (60.6)	9681	1,532,638 (62.0)	10,323
75–84	452,582 (29.8)	16,577	564,958 (32.8)	17,345	640,489 (31.7)	18,159	728,802 (29.5)	17,059
85–94	92,325 (6.1)	8692	108,544 (6.3)	9182	151,473 (7.5)	11,764	202,105 (8.2)	14,109
95+	3191 (0.2)	439	4508 (0.3)	598	5957 (0.3)	830	7820 (0.3)	1057
**Marital status**								
Married	106,3745 (70.1)	18,728	1,196,496 (69.4)	18,314	1,382,643 (68.3)	20,107	1,635,320 (66.2)	20,494
Widowed	195,044 (12.9)	9925	200,089 (11.6)	9428	211,086 (10.4)	9786	229,634 (9.3)	9518
Divorced	122,780 (8.1)	3354	171,712 (10.0)	4271	243,798 (12.0)	5255	350,024 (14.2)	6433
Never married	136,216 (9.0)	4577	155,892 (9.0)	4857	185,939 (9.2)	5286	256,388 (10.4)	6103
**Education**								
High	231,804 (15.3)	3962	319,244 (18.5)	4662	460,772 (22.8)	5818	643,907 (26.1)	7345
Intermediate	211,666 (13.9)	4069	303,244 (17.6)	4967	450,551 (22.3)	6493	695,875 (28.2)	8644
Low	1,074,315 (70.8)	28,553	1,101,701 (63.9)	27,241	1,112,143 (55.0)	28,123	1,131,583 (45.8)	26,559
Total	100.0		100.0		100.0		100.0	
n	1,517,785	36,584	1,724,190	36,870	2,023,465	40,434	2,471,365	42,548
**Women**								
Age								
65–74	1,221,805 (50.8)	12,300	1,244,423 (48.6)	9898	1,399,911 (49.8)	9197	1,701,105 (52.8)	8979
75–84	881,296 (36.6)	35,511	967,286 (37.8)	32,690	980,689 (34.9)	30,031	1,022,598 (31.8)	25,420
85–94	287,140 (11.9)	29,088	324,941 (12.7)	27,879	399,964 (14.2)	32,725	460,583 (14.3)	34,860

95+	15,051 (0.6)	2075	21,785 (0.9)	2515	28,543 (1.0)	3620	35,025 (1.1)	4152
**Marital status**								
Married	872,851 (36.3)	13,105	994,851 (38.9)	13,444	1,166,532 (41.5)	14,613	1,409,637 (43.8)	14,903
Widowed	1,055,960 (43.9)	48,828	1,026,337 (40.1)	43,747	998,641 (35.6)	43,814	983,439 (30.5)	40,738
Divorced	228,436 (9.5)	6769	295,148 (11.5)	7021	393,316 (14.0)	8354	537,422 (16.7)	9810
Never married	248,045 (10.3)	10,272	242,098 (9.5)	8770	250,618 (8.9)	8792	288,813 (9.0)	7960
**Education**								
High	214,921 (8.9)	4920	300,441 (11.7)	5287	453,189 (16.1)	6835	676,688 (21.0)	8317
Intermediate	366,008 (15.2)	9072	481,994 (18.8)	10,024	653,707 (23.3)	12,486	928,022 (28.8)	14,628
Low	1,824,363 (75.8)	64,982	1,776,000 (69.4)	57,671	1,702,211 (60.6)	56,252	1,614,601 (50.2)	50,466
Total	100.0		100.0		100.0		100.0	
n	2,405,292	78,974	2,558,435	72,982	2,809,107	75,573	3,219,311	73,411

Abbreviation: LTC, residential long-term care

Supplementary Figure 1. Age-specific annual transition probabilities from (A) home to residential long-term care (LTC), (B) home to death, (C) LTC to home, and (D) LTC to death by period, men

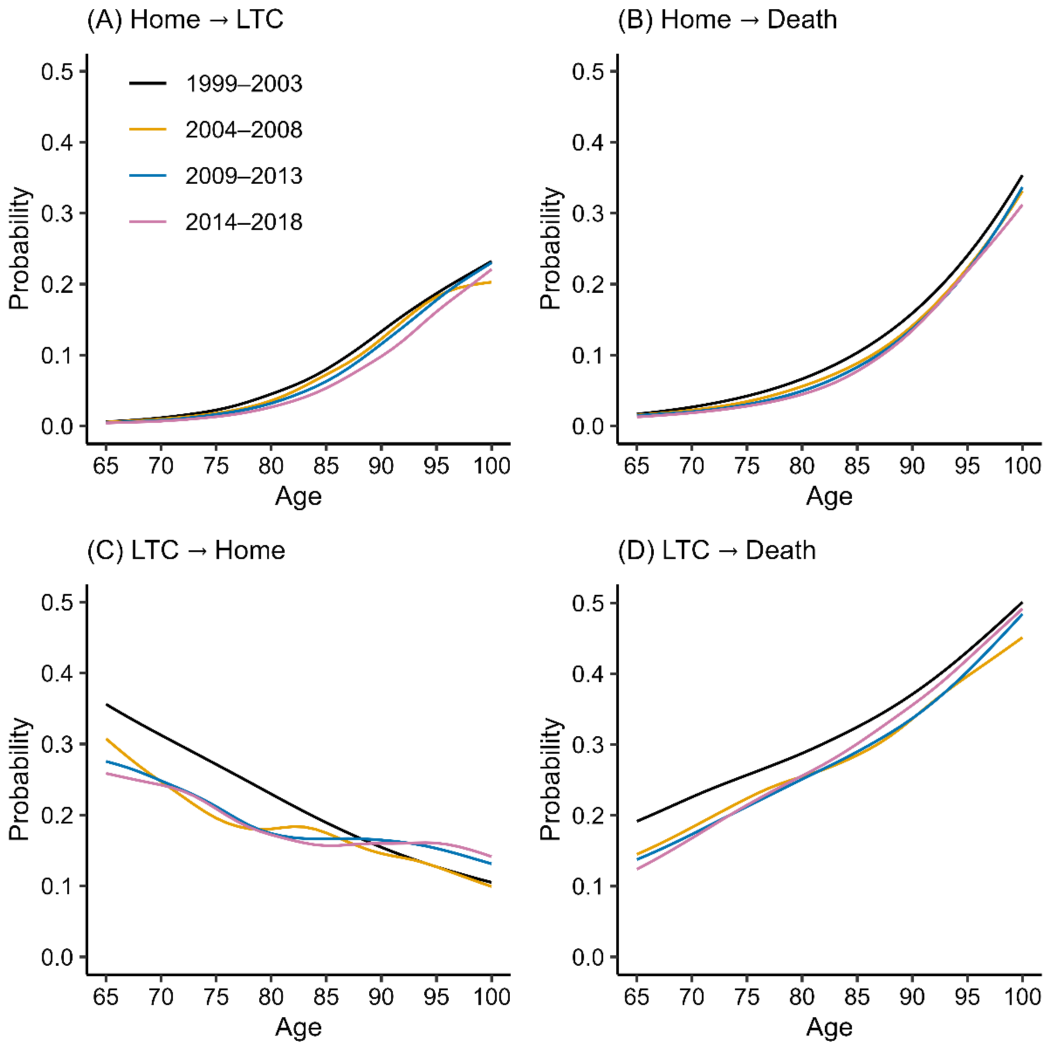

Note: Transition probabilities are shown only up to age 100 years because estimates at older ages are based on small numbers and their publication is prohibited due to data protection regulations.

Supplementary Figure 2. Age-specific annual transition probabilities from (A) home to residential long-term care (LTC), (B) home to death, (C) LTC to home, and (D) LTC to death by period, women

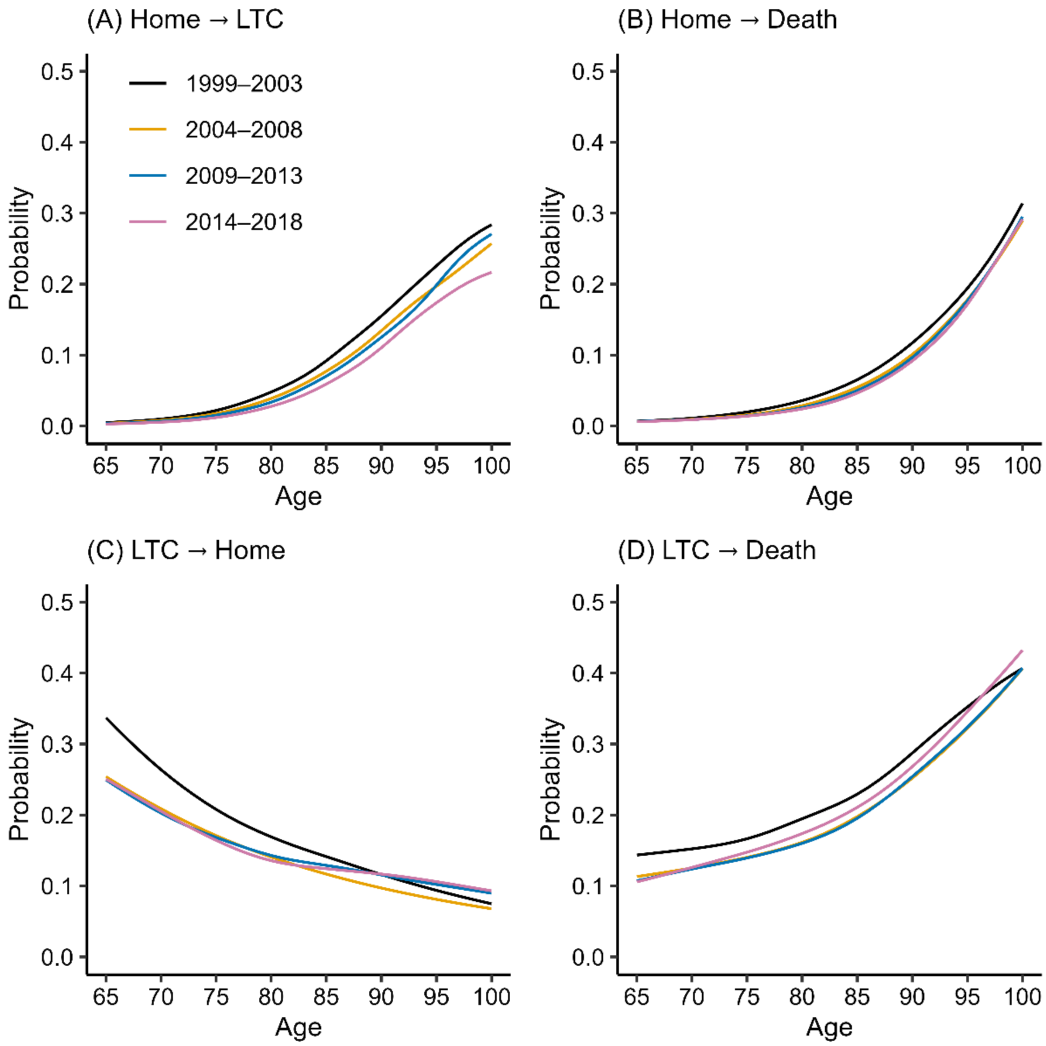

Note: Transition probabilities are shown only up to age 100 years because estimates at older ages are based on small numbers and their publication is prohibited due to data protection regulations.

Supplementary Table 2. Total life expectancy at age 65 years in 1999–2018, estimated from the multistate model and the Official Statistics of Finland,[1] by sex

Year	Men		Women	
	Multistate model	Official Statistics of Finland	Multistate model	Official Statistics of Finland
1999	15.10 (14.99–15.21)	15.15	19.27 (19.18–19.37)	19.30
2000	15.49 (15.37–15.61)	15.46	19.38 (19.28–19.49)	19.37
2001	15.71 (15.59–15.82)	15.70	19.68 (19.58–19.77)	19.66
2002	15.78 (15.67–15.88)	15.76	19.71 (19.61–19.81)	19.67
2003	16.15 (16.04–16.26)	16.14	19.94 (19.84–20.04)	19.87
2004	16.50 (16.38–16.61)	16.46	20.57 (20.47–20.67)	20.48
2005	16.77 (16.65–16.88)	16.73	20.81 (20.71–20.91)	20.71
2006	16.80 (16.69–16.91)	16.77	20.93 (20.82–21.03)	20.89
2007	16.94 (16.83–17.05)	16.90	21.07 (20.97–21.17)	20.95
2008	17.39 (17.28–17.51)	17.30	21.24 (21.14–21.33)	21.03
2009	17.25 (17.13–17.36)	17.16	21.34 (21.23–21.44)	21.19
2010	17.32 (17.21–17.44)	17.30	21.23 (21.14–21.33)	21.16
2011	17.61 (17.50–17.72)	17.55	21.49 (21.39–21.59)	21.37
2012	17.72 (17.62–17.84)	17.61	21.50 (21.40–21.60)	21.27
2013	17.95 (17.84–18.06)	17.81	21.70 (21.61–21.80)	21.49
2014	18.06 (17.96–18.17)	17.97	21.65 (21.55–21.75)	21.45
2015	18.22 (18.11–18.32)	18.09	21.79 (21.70–21.88)	21.57
2016	18.12 (18.02–18.22)	18.04	21.83 (21.73–21.92)	21.59
2017	18.32 (18.22–18.42)	18.23	21.91 (21.82–22.01)	21.76
2018	18.45 (18.35–18.55)	18.36	22.04 (21.95–22.13)	21.83

Supplementary Table 3. Total life expectancy and life expectancy in LTC[a] at age 65 years by marital status and educational level, and by period and sex

	1999–2003		2004–2008		2009–2013		2014–2018	
	**Total life expectancy**	**Care expectancy**	**Total life expectancy**	**Care expectancy**	**Total life expectancy**	**Care expectancy**	**Total life expectancy**	**Care expectancy**
**Men**								
All	15.65 (15.61–15.70)	0.75 (0.74–0.76)	16.89 (16.84–16.94)	0.89 (0.88–0.90)	17.58 (17.54–17.63)	0.87 (0.86–0.89)	18.24 (18.19–18.29)	0.8 (0.79–0.81)
**Marital status**								
Married	16.58 (16.52–16.65)	0.60 (0.58–0.61)	18.05 (17.98–18.12)	0.72 (0.70–0.74)	18.77 (18.70–18.83)	0.71 (0.70–0.73)	19.54 (19.48–19.59)	0.67 (0.66–0.69)
Widowed	14.46 (14.29–14.62)	0.86 (0.83–0.89)	15.47 (15.28–15.65)	1.02 (0.98–1.05)	16.18 (15.99–16.34)	0.98 (0.94–1.01)	16.81 (16.64–16.97)	0.87 (0.84–0.89)
Divorced	13.28 (13.12–13.44)	0.96 (0.92–1.01)	14.30 (14.15–14.47)	1.10 (1.05–1.16)	15.22 (15.08–15.36)	1.09 (1.05–1.13)	15.97 (15.87–16.09)	0.94 (0.91–0.98)
Never married	13.04 (12.88–13.20)	1.19 (1.15–1.25)	13.87 (13.71–14.02)	1.35 (1.31–1.41)	14.48 (14.34–14.62)	1.27 (1.22–1.31)	15.21 (15.08–15.34)	1.16 (1.12–1.19)
**Education**								
High	17.71 (17.58–17.86)	0.70 (0.67–0.74)	19.01 (18.88–19.17)	0.86 (0.82–0.90)	19.60 (19.49–19.73)	0.84 (0.81–0.87)	20.15 (20.07–20.27)	0.74 (0.71–0.76)
Intermediate	16.37 (16.20–16.49)	0.72 (0.70–0.76)	17.34 (17.18–17.45)	0.90 (0.87–0.94)	17.83 (17.68–17.93)	0.88 (0.86–0.93)	18.33 (18.22–18.41)	0.78 (0.76–0.81)
Low	15.15 (15.07–15.18)	0.76 (0.76–0.78)	16.26 (16.17–16.30)	0.90 (0.90–0.93)	16.81 (16.72–16.85)	0.89 (0.88–0.91)	17.29 (17.22–17.34)	0.83 (0.82–0.85)
**Women**								
All	19.60 (19.55–19.64)	1.61 (1.59–1.62)	20.93 (20.88–20.97)	1.83 (1.81–1.85)	21.45 (21.41–21.5)	1.74 (1.72–1.75)	21.85 (21.8–21.89)	1.51 (1.50–1.53)
**Marital status**								
Married	20.34 (20.24–20.45)	1.32 (1.27–1.37)	21.84 (21.72–22.02)	1.66 (1.59–1.79)	22.51 (22.38–22.78)	1.64 (1.56–1.88)	23.02 (22.91–23.17)	1.46 (1.40–1.55)
Widowed	19.40 (19.32–19.47)	1.63 (1.61–1.65)	20.65 (20.58–20.73)	1.87 (1.84–1.89)	21.09 (21.00–21.17)	1.79 (1.76–1.81)	21.39 (21.30–21.47)	1.56 (1.54–1.58)
Divorced	18.61 (18.46–18.76)	1.80 (1.75–1.86)	19.93 (19.79–20.06)	1.98 (1.92–2.04)	20.49 (20.36–20.63)	1.85 (1.80–1.91)	20.82 (20.71–20.94)	1.60 (1.56–1.64)
Never married	18.40 (18.26–18.55)	1.92 (1.88–1.97)	19.57 (19.41–19.73)	2.19 (2.13–2.24)	19.95 (19.79–20.08)	2.10 (2.04–2.15)	20.18 (20.04–20.31)	1.77 (1.72–1.82)
**Education**								

High	21.18 (21.02–21.35)	1.67 (1.61–1.74)	22.62 (22.48–22.79)	1.96 (1.88–2.03)	22.89 (22.76–23.03)	1.77 (1.71–1.83)	23.38 (23.27–23.50)	1.55 (1.50–1.59)
Intermediate	20.32 (20.19–20.43)	1.64 (1.59–1.69)	21.54 (21.42–21.65)	1.86 (1.81–1.91)	21.89 (21.77–21.97)	1.74 (1.71–1.79)	22.17 (22.06–22.24)	1.55 (1.53–1.60)
Low	19.32 (19.24–19.35)	1.60 (1.59–1.62)	20.55 (20.48–20.59)	1.82 (1.81–1.85)	21.00 (20.94–21.05)	1.75 (1.73–1.77)	21.17 (21.12–21.23)	1.52 (1.50–1.54)

[a] Years of total life expectancy at age 65 years spent in residential long-term care

Supplementary Table 4. Proportion of 65-year-olds ever entering LTC, median age at first entry, and years in LTC if entered, by marital status and educational level, and by period and sex

	1999–2003			2004–2008			2009–2013			2014–2018		
	% ever entering	Median age	Years in LTC	% ever entering	Median age	Years in LTC	% ever entering	Median age	Years in LTC	% ever entering	Median age	Years in LTC
**Men**												
All	33.5 (33.2–33.8)	80.9 (80.8–81.0)	2.24 (2.21–2.27)	35.1 (34.8–35.4)	82.2 (82.1–82.3)	2.53 (2.49–2.58)	35.1 (34.8–35.4)	83.0 (82.9–83.1)	2.49 (2.45–2.53)	33.8 (33.5–34.1)	84.0 (83.9–84.1)	2.37 (2.33–2.40)
**Marital status**												
Married	31.1 (30.6–31.5)	82.4 (82.2–82.5)	1.93 (1.88–1.98)	33.2 (32.7–33.7)	84.0 (83.8–84.1)	2.17 (2.11–2.24)	33.4 (32.9–33.8)	84.7 (84.5–84.8)	2.14 (2.08–2.20)	32.4 (32.0–32.9)	85.6 (85.4–85.7)	2.08 (2.03–2.14)
Widowed	35.9 (35.1–36.7)	79.1 (78.8–79.4)	2.39 (2.30–2.48)	37.1 (36.2–37.8)	79.9 (79.6–80.2)	2.74 (2.64–2.85)	36.4 (35.6–37.3)	81.1 (80.8–81.4)	2.68 (2.57–2.78)	34.2 (33.4–34.9)	82.0 (81.7–82.3)	2.53 (2.44–2.63)
Divorced	36.7 (35.6–37.8)	77.2 (76.8–77.5)	2.63 (2.48–2.79)	37.9 (36.9–39.1)	78.3 (77.9–78.6)	2.90 (2.75–3.07)	36.8 (35.8–37.7)	79.7 (79.4–80.0)	2.95 (2.83–3.10)	35.1 (34.2–36.0)	81.0 (80.7–81.3)	2.68 (2.57–2.81)
Never married	38.7 (37.8–39.7)	75.8 (75.5–76.1)	3.08 (2.94–3.24)	39.3 (38.4–40.3)	76.5 (76.2–76.8)	3.44 (3.30–3.60)	38.8 (37.9–39.7)	77.9 (77.6–78.3)	3.26 (3.13–3.41)	38.0 (37.2–38.9)	79.0 (78.7–79.3)	3.05 (2.91–3.19)
**Education**												
High	33.9 (32.9-34.9)	83.1 (82.8–83.4)	2.07 (1.98–2.19)	36.6 (35.6-37.5)	84.4 (84.2–84.7)	2.35 (2.24–2.48)	35.7 (34.8-36.6)	85.0 (84.8–85.3)	2.36 (2.26–2.46)	34.4 (33.6-35.1)	85.8 (85.5–86.0)	2.16 (2.06–2.24)
Intermediate	34.5 (33.6-35.5)	81.6 (81.2–81.9)	2.08 (1.99–2.22)	36.5 (35.5-37.5)	82.9 (82.6–83.2)	2.46 (2.36–2.61)	35.3 (34.5-36.3)	83.4 (83.1–83.7)	2.49 (2.41–2.64)	33.8 (33.0-34.5)	84.1 (83.9–84.4)	2.31 (2.23–2.42)
Low	33.2 (32.9-33.6)	80.3 (80.2–80.4)	2.29 (2.27–2.36)	34.7 (34.4-35.1)	81.4 (81.3–81.6)	2.59 (2.58–2.68)	34.8 (34.5-35.2)	82.2 (82.1–82.3)	2.55 (2.52–2.62)	33.5 (33.2-33.9)	82.9 (82.8–83.0)	2.47 (2.43–2.54)
**Women**												
All	51.3 (51.0–51.6)	83.3 (83.2–83.3)	3.14 (3.10–3.17)	52.3 (52.0–52.6)	84.5 (84.4–84.6)	3.50 (3.46–3.53)	51.6 (51.3–51.9)	85.3 (85.3–85.4)	3.37 (3.33–3.40)	48.9 (48.6–49.2)	86.2 (86.1–86.3)	3.09 (3.05–3.13)
**Marital status**												
Married	50.1 (49.1–51.1)	84.6 (84.4–84.8)	2.63 (2.52–2.73)	51.9 (50.7–53.0)	85.8 (85.6–86.0)	3.19 (3.05–3.48)	52.2 (51.0–53.4)	86.8 (86.5–86.9)	3.15 (2.98–3.59)	50.1 (49.1–51.2)	87.6 (87.4–87.8)	2.91 (2.77–3.10)
Widowed	51.4 (51.0–51.8)	82.9 (82.8–83.0)	3.17 (3.13–3.21)	52.5 (52.1–52.9)	84.1 (84.0–84.2)	3.55 (3.50–3.61)	51.8 (51.4–52.2)	84.9 (84.7–85.0)	3.45 (3.39–3.50)	49.0 (48.5–49.4)	85.7 (85.6–85.8)	3.19 (3.14–3.24)
Divorced	53.3 (52.3–54.2)	81.9 (81.7–82.1)	3.38 (3.26–3.51)	53.0 (52.0–54.0)	83.4 (83.2–83.6)	3.74 (3.61–3.89)	51.4 (50.4–52.2)	84.2 (84.0–84.5)	3.60 (3.49–3.73)	48.6 (47.8–49.5)	85.1 (84.9–85.3)	3.29 (3.18–3.41)

Never married	54.0 (53.2–54.8)	81.2 (81.0–81.4)	3.56 (3.46–3.67)	55.3 (54.5–56.2)	82.2 (82.0–82.4)	3.96 (3.83–4.07)	55.1 (54.3–55.9)	82.9 (82.7–83.1)	3.81 (3.68–3.92)	50.7 (49.8–51.5)	84.0 (83.8–84.2)	3.49 (3.37–3.62)
**Education**												
High	53.4 (52.3-54.5)	84.6 (84.4–84.9)	3.13 (3.02–3.25)	54.2 (53.0-55.4)	86.0 (85.8–86.2)	3.61 (3.45–3.76)	54.3 (53.3-55.3)	86.8 (86.6–87.0)	3.26 (3.14–3.39)	50.6 (49.6-51.5)	87.6 (87.4–87.7)	3.05 (2.94–3.17)
Intermediate	52.6 (51.8-53.5)	84.0 (83.8–84.2)	3.11 (3.01–3.22)	53.6 (52.7-54.4)	85.0 (84.8–85.1)	3.47 (3.38–3.58)	52.3 (51.6-53.0)	85.7 (85.5–85.9)	3.32 (3.25–3.42)	49.7 (49.0-50.4)	86.5 (86.4–86.7)	3.12 (3.06–3.23)
Low	51.0 (50.7-51.3)	83.0 (82.9–83.1)	3.14 (3.12–3.19)	51.9 (51.6-52.3)	84.2 (84.1–84.2)	3.51 (3.48–3.56)	51.2 (50.8-51.5)	84.9 (84.9–85.0)	3.41 (3.37–3.46)	48.3 (48.0-48.7)	85.7 (85.6–85.8)	3.14 (3.09–3.19)

Abbreviation: LTC, residential long-term care

Supplementary Figure 3. Contributions of changes in transition rates between home, residential long-term care (LTC), and death to the change in life expectancy in LTC at age 65 years[a] between 1999–2003, 2004–2008, 2009–2013 and 2014–2018, by marital status, men

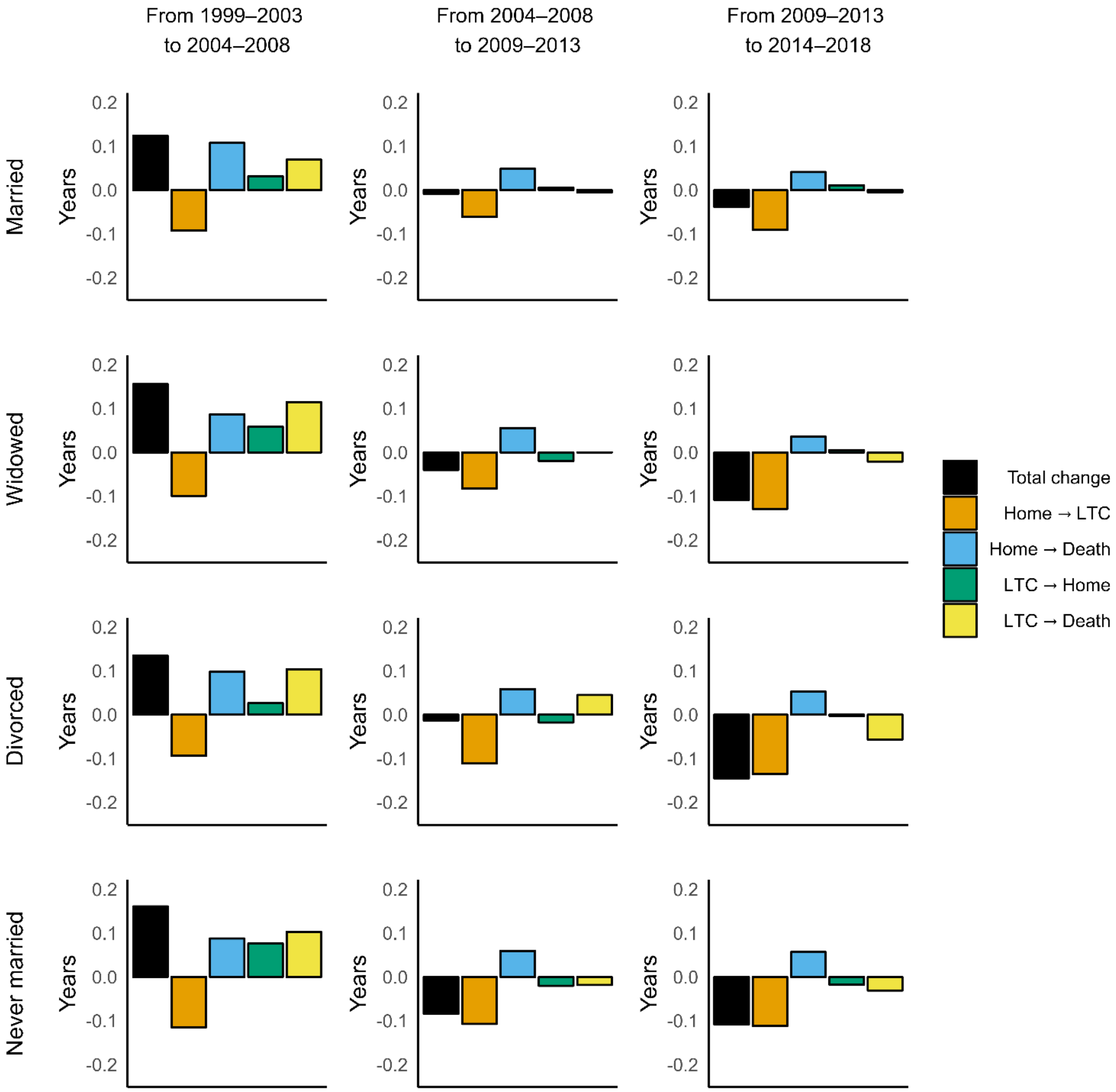

[a] Years of total life expectancy at age 65 years spent in LTC.

Supplementary Figure 4. Contributions of changes in transition rates between home, residential long-term care (LTC), and death to the change in life expectancy in LTC at age 65 years[a] between 1999–2003, 2004–2008, 2009–2013 and 2014–2018, by marital status, women

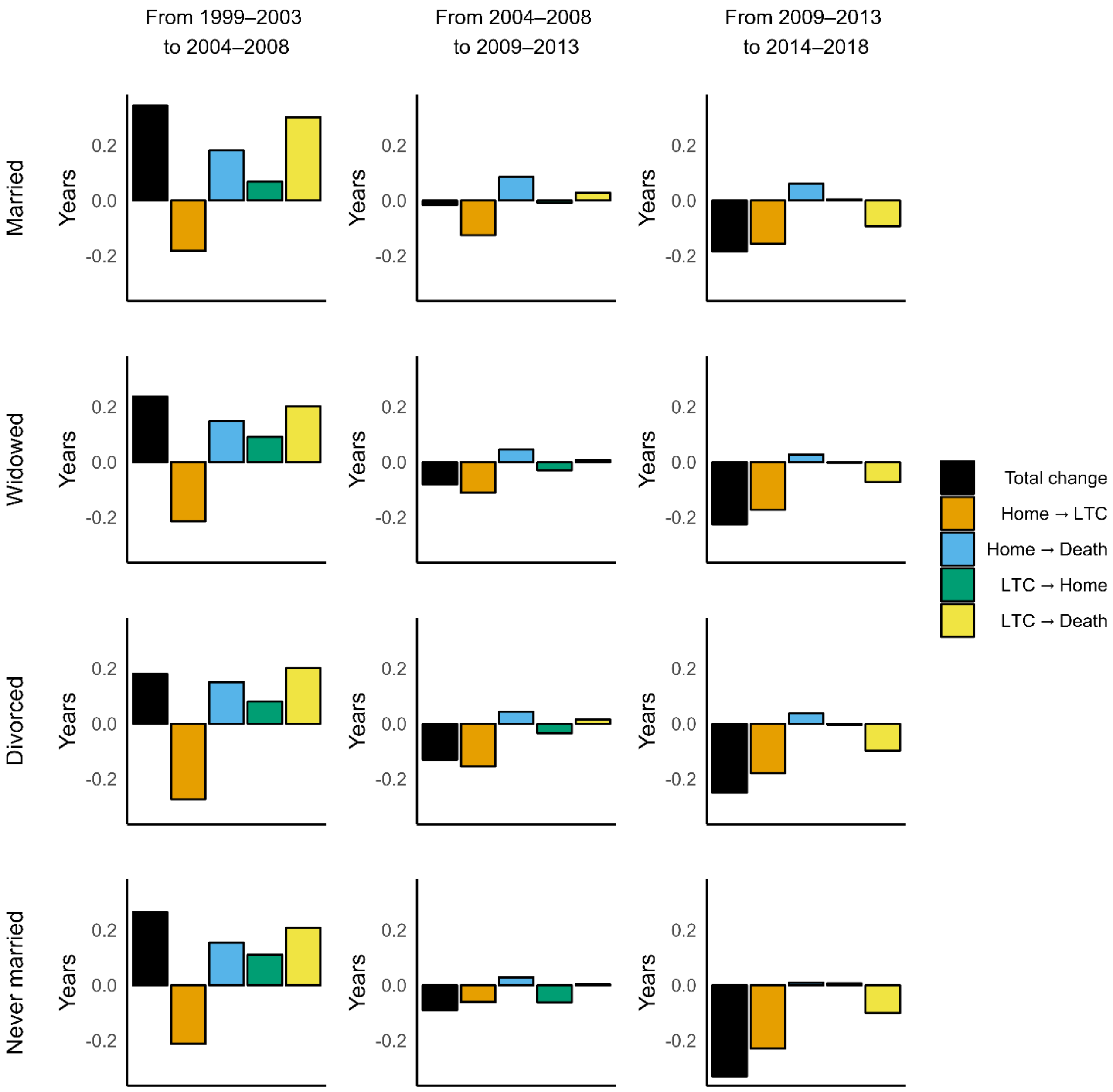

[a] Years of total life expectancy at age 65 years spent in LTC.

Supplementary Figure 5. Contributions of changes in transition rates between home, residential long-term care (LTC), and death to the change in life expectancy in LTC at age 65 years[a] between 1999–2003, 2004–2008, 2009–2013 and 2014–2018, by educational level, men

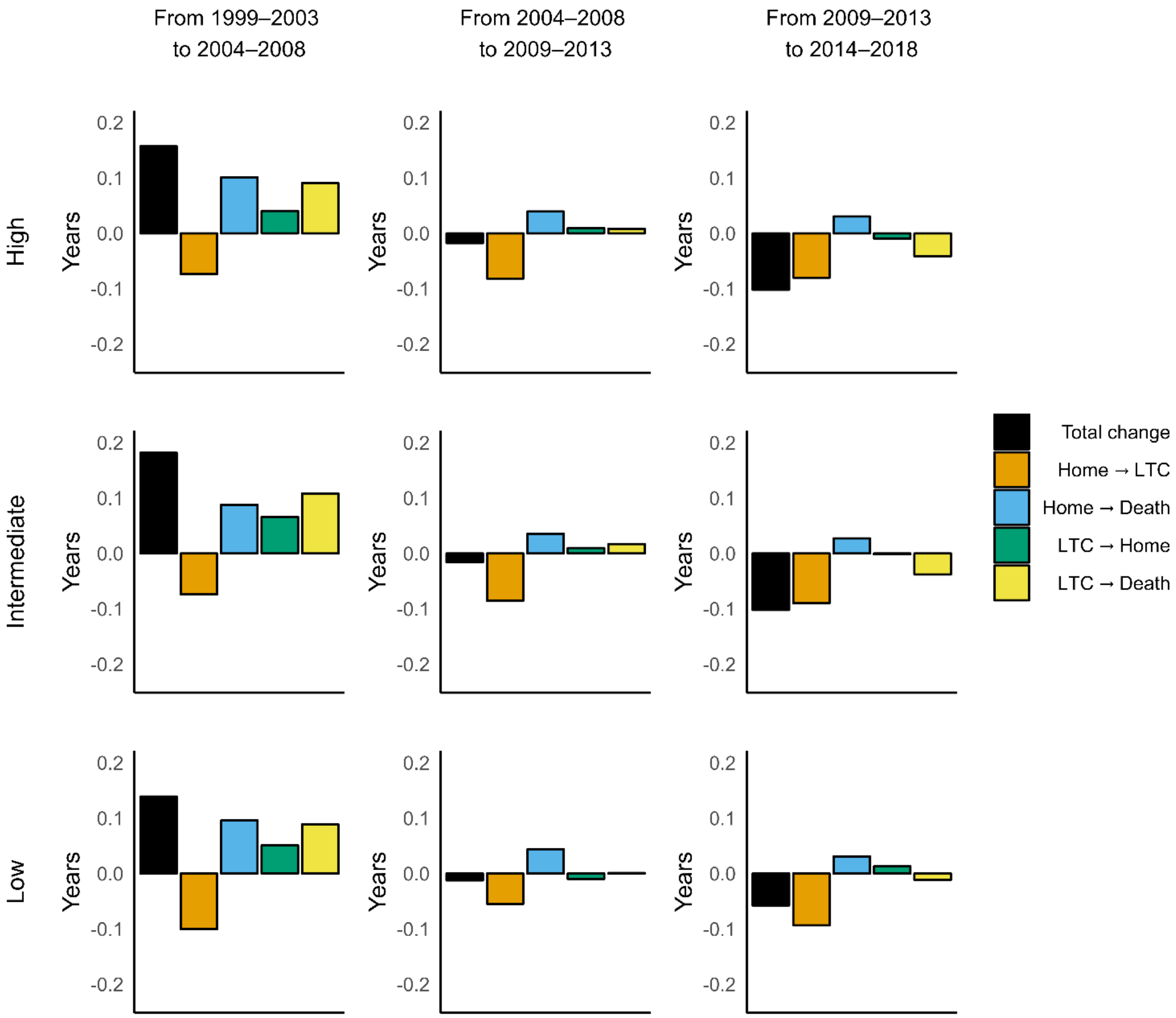

[a] Years of total life expectancy at age 65 years spent in LTC.

Supplementary Figure 6. Contributions of changes in transition rates between home, residential long-term care (LTC), and death to the change in life expectancy in LTC at age 65 years[a] between 1999–2003, 2004–2008, 2009–2013 and 2014–2018, by educational level, women

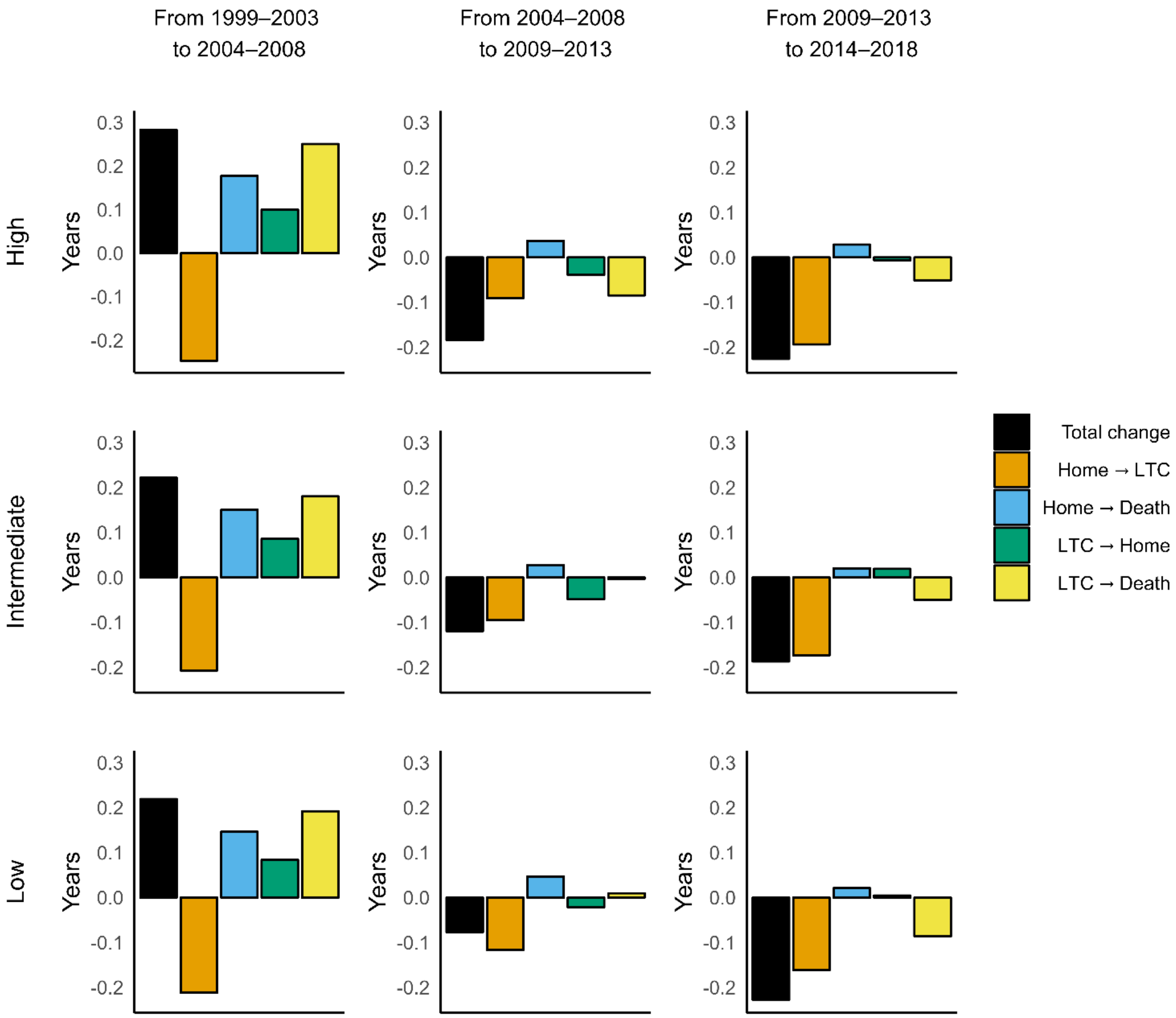

[a] Years of total life expectancy at age 65 years spent in LTC.

Supplementary Figure 7. Life expectancy in LTC at age 65 years[a], by area of residence and period, and by sex

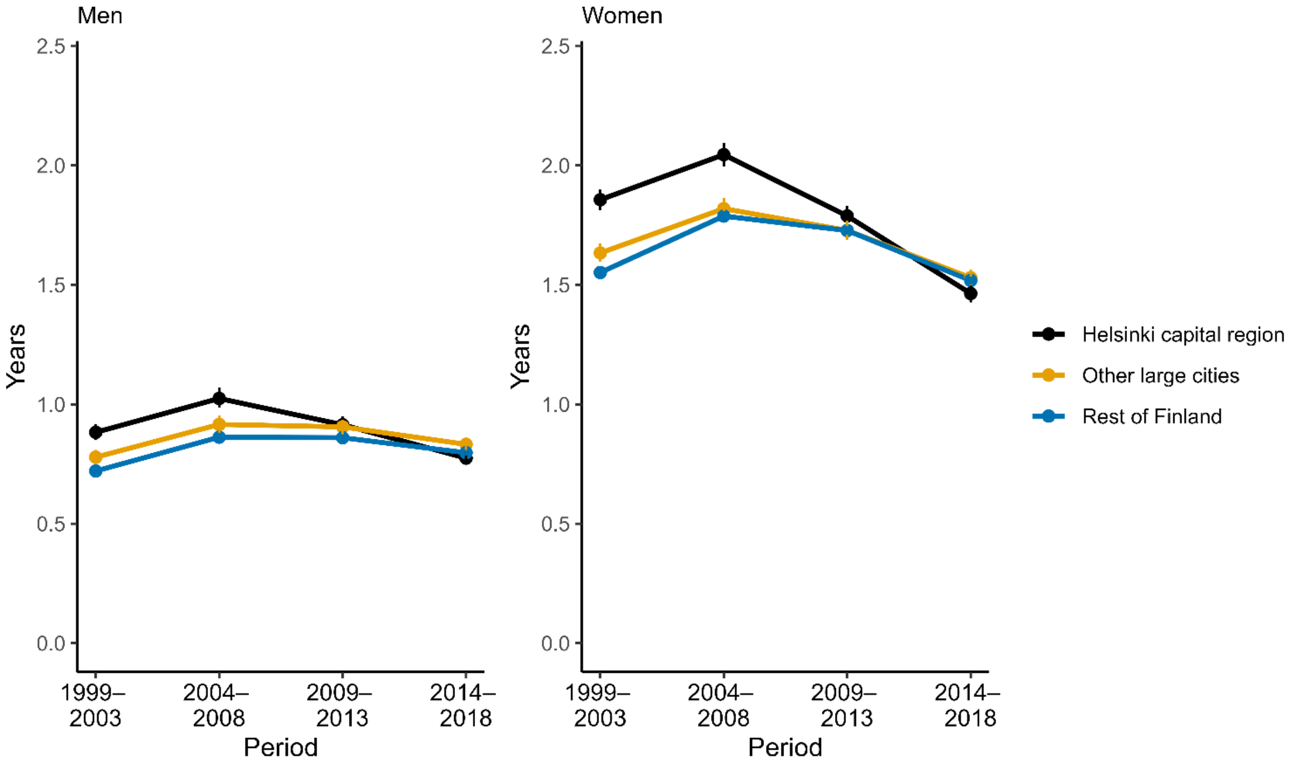

[a] Years of total life expectancy at age 65 years spent in residential long-term care.

Supplementary Figure 8. Contributions of changes in transition rates between home, residential long-term care (LTC), and death to the change in life expectancy in LTC at age 65 years[a] between 1999–2003, 2004–2008, 2009–2013 and 2014–2018, by area of residence, men

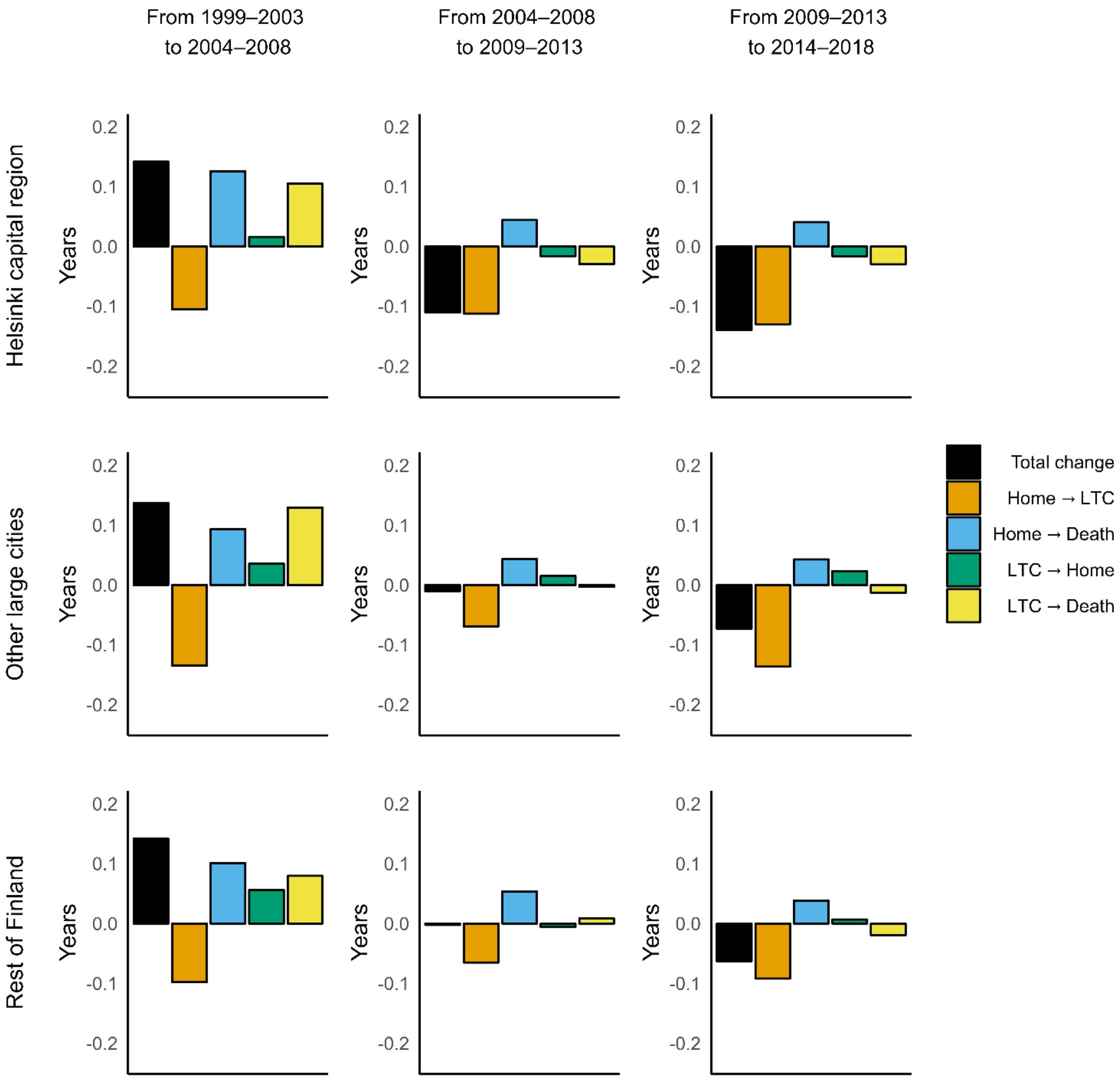

[a] Years of total life expectancy at age 65 years spent in LTC.

Supplementary Figure 9. Contributions of changes in transition rates between home, residential long-term care (LTC), and death to the change in life expectancy in LTC at age 65 years[a] between 1999–2003, 2004–2008, 2009–2013 and 2014–2018, by area of residence, women

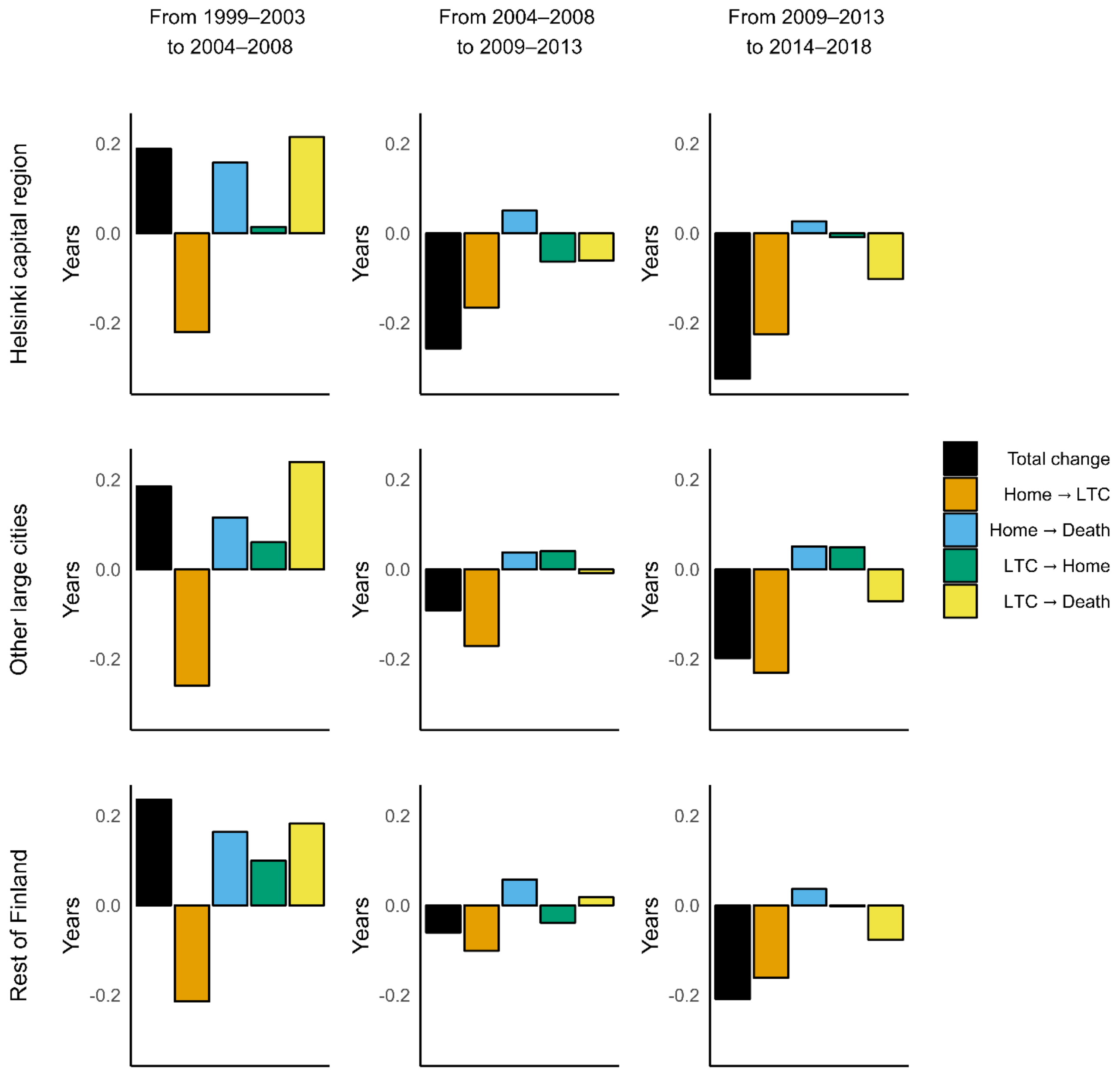

[a] Years of total life expectancy at age 65 years spent in LTC.

References:

1 Life expectancy, years, 2022. Official Statistics of Finland (OSF). Available at: *https://pxdata.stat.fi:443/PxWeb/sq/ebece6d1-67f9-4c80-98bf-88eed249fb86*. Accessed Jul 11, 2023.
